# *Coptidis rhizome* and Si Jun Zi Tang Can Prevent *Salmonella enterica* Serovar Typhimurium Infection in Mice

**DOI:** 10.1371/journal.pone.0105362

**Published:** 2014-08-18

**Authors:** Chiung-Hung Chang, Bi Yu, Chiu-Hsian Su, Daniel S. Chen, Yu-Chi Hou, Yueh-Sheng Chen, Yuan-Man Hsu

**Affiliations:** 1 Graduate Institute of Chinese Medicine, School of Chinese Medicine, China Medical University, Taichung, Taiwan; 2 Department of Traditional Chinese Medicine, Taichung Veterans General Hospital, Taichung, Taiwan; 3 Department of Animal Science, College of Agriculture and Natural Resources, National Chung Hsing University, Taichung, Taiwan; 4 Department of Biological Science and Technology, College of Life Sciences, China Medical University, Taichung, Taiwan; 5 Department of Biochemistry, College of Agriculture and Life Sciences, University of Wisconsin at Madison, Madison, Wisconsin, United States of America; 6 Department of Pharmacy, College of Pharmacy, China Medical University, Taichung, Taiwan; 7 Department of Traditional Chinese Medicine, Tainan Municipal Hospital, Tainan City, Taiwan; Rochester Institute of Technology, United States of America

## Abstract

*Salmonella*, a common zoonotic pathogen, causes gastroenteritis in both humans and animals. Traditional Chinese Medicine (TCM) has been used to improve gastrointestinal dysfunction and to modify the immune response to inflammation for centuries. This study used six herbal plants and four TCM formulae to rate their efficacy in preventing *S.* Typhimurium infection via mouse model. Minimum bactericidal concentration (MBC) of *Coptidis rhizome* (CR) against the reference strain tallied 12.5 mg/ml and against clinical isolate ST21 was 25 mg/ml. MBCs of other herbal extracts and formulae on *Salmonella* Typhimurium strains were above 50 mg/ml. In the mice model, CR and Si Jun Zi Tang (SJZT) could significantly decrease the bacterial load in organs and blood after being challenged, along with body weight loss due to the infection. CR and SJZT alleviated infection-induced interferon-gamma levels in the serum and tissues, and tumor necrosis factor-alpha (TNF-α) levels in intestinal tissues. CR and SJZT serum metabolites could suppress *S.* Typhimurium invasion and TNF-α expression in RAW264.7 cells. The therapeutic activity of CR and SJZT may involve berberine, ginsenoside Rb1, and glycyrrhizin, interfering with *Salmonella* when invading macrophages. CR and SJZT has shown potential in preventing *S.* Typhimurium infection through the regulation of the immune response.

## Introduction

*Salmonella* causes salmonellosis in humans and severe infections in animals [1], which constitutes major public health problems, creating a severe economic impact on many countries. Over 2,500 *Salmonella* serotypes were identified up to 2004 [2]. Among them, *Salmonella enterica* is an important pathogen responsible for food-borne diseases. *S. enterica* serovar Typhimurium is a key serotype for salmonellosis transmitted from animals to humans [1]. Gastroenteritis caused by a *Salmonella* infection is usually self-limited, where antimicrobial treatment is seldom required [3]. Still, bacteria passing through the intestines and entering the bloodstream do warrant antibiotic treatment. Antibiotic-resistant strains have arisen; over 50% of the isolates proved resistant to ampicillin, chloramphenicol, and trimethoprim/sulfamethoxazole, the primary treatments of choice for salmonellosis [4]–[7]. Use of antibiotics in animals might result in the deposition of residues in meat products, which elevates the risk of spawning more resistant strains [4], [8]–[10]; herbal substitutes for antibiotics hold potential for the prevention and/or control of this disease.

Antibiotic treatment of infectious disease raises controversy worldwide. Utilizing the various traits of herbal plants in order to replace antibiotics might offer an alternative. Herbs that manifest certain qualities inhibiting *Salmonella* growth: e.g., extracts of CR and *Gardeniae fructus*, can exhibit antibacterial activity against certain strains, and the aqueous extract of *Schizandrae fructus* can be used to suppress growth of *S.* Paratyphi and *S.* Gallinarum [11]. It has been demonstrated that a methanol extract of CR, *Mume fructus*, and *Scutellariae radix* combination, offered an alternative way to treat *S.* Gallinarum infection in chickens [12]. The aqueous extract of *Houttuyniae herba* has been shown to inhibit *S.* Typhimurium growth and its invasion of macrophages; it can also significantly increase the survival time of infected mice [13]. Many TCM formulas have been widely used for improving gastrointestinal dysfunction and modifying the immune response to inflammation, such as Ger Gen Chyn Lian Tang and Huoh Shiang Jeng Chih San, which have shown curative effects in treating infectious diseases [14], [15]. Shen Ling Bai Zhu San has been reported to be capable of eliminating discomfort when diagnosed with irritable bowel syndrome and diarrhea [16], [17]. In a prior study, SJZT has even been shown to increase phagocytosis of mouse peritoneal macrophages [18], [19]. Yet there are not many studies that conduct animal infection models to verify the bioactivities of these herbal materials. Herbal compounds that are administered orally, go through different metabolic systems or are altered through biotransformation. The bioactivities of herbal compounds in *in vivo* might be quite different when used in *in vitro*. In this study, six herbal plants and four traditional Chinese medicine (TCM) formulae were used to evaluate their efficiency in preventing *S.* Typhimurium infection, using a murine model. *Coptidis rhizome* (CR), *Houttuyniae herba, Taraxaci herba, Glycyrrhizae radix, Puerariae radix*, and *Rhizoma dioscoreae* are herbal plants with antibacterial potential, used in Taiwanese folk medicine; Shen Ling Bai Zhu San and Si Jun Zi Tang (SJZT) are TCM formulas used for adjusting immunity [18], [19]; Huoh Shiang Jeng Chih San and Ger Gen Chyn Lian Tang are remedies for gastrointestinal dysfunction [14], [15]. This study offers an alternative way to diminish the risk of *Salmonella* infection in humans and animals and the further development of multidrug-resistant strains by utilizing various characteristics of these herbal plants.

## Materials and Methods

### Ethic Statement

Male BALB/c mice weighing about 20–22 g, obtained from the National Laboratory for Animal Breeding and Research Center, were maintained in specific pathogen-free (SPF) condition and used at 8–10 weeks of age. All were housed in an air-conditioned room at 25±2 °C with a relative humidity of 40–70%, a 12-hr light/dark cycle, and fed with tap water and the standard laboratory rodent diet. All animal experimental protocols were approved by the Institutional Animal Care and Use Committee of China Medical University (approved number: 99-156-B), experiments performed according to institutional ethical rules and laws. Mouse were humanely euthanized by isofluorane before we collected blood and organs. All efforts were made to minimize suffering.

### Preparation of herbal extracts, TCM formulas and indicator compounds

All herbal materials were purchased from Ko Da Pharmaceutical Co., Ltd. in Taoyuan, Taiwan. Their origins were identified macroscopically and microscopically by the company. Six herbs used in this study were *Coptidis rhizoma* (CR), *Houttuyniae herba, Taraxaci herba, Glycyrrhizae radix, Puerariae radix*, and *Rhizoma dioscoreae*; the four TCM formulas used were Huoh Shiang Jeng Chih San, Ger Gen Chyn Lian Tang, Shen Ling Bai Zhu San, and Si Jun Zi Tang (SJZT), which were all prepared according to original Chinese documents at ratios listed in Table 1. Extracts were prepared as described below. Plant materials were finely powdered and extracted with distilled water at 100°C for 1 hr (plant: water  = 1∶10, w/v). After filtering out insoluble matter, the filtrate was concentrated in a vacuum and lyophilized to yield residue. The percentage of indicator compound in each original herb as well as TCM formula was confirmed by high-performance liquid chromatogram(HPLC) by Ko Da Pharmaceutical Co., Ltd. (Table 2). Indicator compounds berberine, ginsenoside Rb1, and glycyrrhizin were purchased from Sigma-Aldrich Co. LLC, (St. Louis, MO) for further *in vitro* tests.

**Table 1:** Herbal components of TCM formulas used in this study.

TCM formula	Herbal component	Ratio by weight
Huoh Shiang Jeng Chih San	*Pericarpium arecae*, *Poria cocos*, *Angelicae dahuricae radix*, *Perillae folium*, *Citri tangerine pericarpium*, *Platycodi radix*, *Atractylodis macrocephalae*, *Magnoliae cortex*, *Pinellia ternata*, *Glycyrrhizae radix*, *Agastachis herba*, *Zingiberis rhizoma*, *Jujubae fructus*	3∶3∶3∶3∶2∶2∶2∶2∶2∶1∶3∶3∶1
Ger Gen Chyn Lian Tang	*Puerariae radix, Glycyrrhizae radix, Scutellariae radix, Coptidis rhizoma*	8∶2∶3∶3
Shen Ling Bai Zhu San	*Lablab album semen, Ginseng radix, Poria cocos, Atractylodis macrocephalae, Glycyrrhizae radix, Rhizoma dioscoreae, Nelumbinis semen, Platycodi radix, Coicis semen, Amomi fructus, Jujubae fructus*	23∶30∶30∶30∶30∶30∶15∶15∶15∶15∶15
Si Jun Zi Tang	*Ginseng radix*, *Poria cocos*, *Glycyrrhizae radix*, *Atractylodis macrocephalae rhizoma*, *Zingiberis rhizoma*, *Jujubae fructus*	6∶6∶3∶6∶3∶2

**Table 2:** Concentrations of indicator compounds in herbal extracts and TCM formulae.

Herbal extract	Indicator compound	mg/g
*Coptidis rhizoma*	Berberine	109.41
*Houttuyniae herba*	Quercetin	0.07
*Taraxaci herba*	Chlorogenic acid	0.18
*Glycyrrhizae radix*	Glycyrrhizic acid	27.913
*Puerariae radix*	Puerarin	29.40
*Rhizoma dioscoreae*	None	-
Huoh Shiang Jeng Chih San	Hesperidin	4.04
	Glycyrrhizin	1.96
Ger Gen Chyn Lian Tang	Baicalin	19.25
	Glycyrrhizin	3.73
Shen Ling Bai Zhu San	Ginsenoside Rb1	1.94
	Glycyrrhizin	3.96
Si Jun Zi Tang	Ginsenoside Rb1	4.33
	Glycyrrhizin	5.18

### Bacterial strains and culture conditions

Reference strain *S.* Typhimurium (ATCC 6994) was purchased from American Type Culture Collection (ATCC). Strain ST21 was collected from a carrier pig and used as inoculum for infecting mice. In order to prevent the antibiotic resistant genes from spreading in the lab animal facility, we chose ST21 which did not show any antibiotic resistance to nalidixic acid, norfloxacin, ciprofloxacin, enrofloxacin, ampicillin, chloramphenicol, streptomycin, sulfamethoxazole, tetracycline, cephalothin, nitrofurantoin, and gentamicin, which are antibiotics widely used for treating infections in the field. Serotype identification of ST21 was applied as follows: Antiserum to detect O and H antigens was purchased from S&A Reagents Lab Limited (Bangkok, Thailand) and Denka Seiken Co., Ltd. (Tokyo, Japan), respectively. The isolate was analyzed according to the Kaufmann-White scheme and serotyping protocols developed by the Centers for Disease Control and Prevention in Atlanta, GA [20]. Bacterial inocula were grown in Luria Bertani (LB) broth to log stationary phase at 250 rpm, 37°C for 8 hr to OD_600_ nm of 0.8. After harvesting by centrifugation at 8,000×g, the bacterial pellet was resuspended in phosphate-buffered saline (PBS), and adjusted to a final concentration of 10^10^ colony forming units (CFU)/ml in PBS.

### Antimicrobial activity

Minimum inhibitory concentration (MIC) of any extract or compound was tested by a two-fold serial dilution. Test extracts were first dissolved in PBS, incorporated into a Muller-Hinton broth at a concentration of 100 mg/ml and then serially diluted to attain 50, 25, 12.5, 6.25, and 3.125 mg/ml; compounds were dissolved in dimethyl sulfoxide (DMSO) to attain a concentration of 20 mM and then serially diluted to 10, 5, 2.5, 1.25, and 0.625 mM. 100 µl of the bacterial suspension, adjusted previously to 10^6^ CFU/ml, was added to 100 µl of an herbal extract or compound, using an additional tube containing only broth was used as the negative control. All test tubes and controls were incubated at 37°C for 18–24 hr, after which the tube containing lowest concentration of extract showing no visible growth was considered as MIC. Further concentrations showing complete inhibition of visual growth of bacteria were identified, with 50 µl of each culture broth transferred onto agar plates and incubated for 18–24 hr at 37°C. Complete absence of growth of bacterial colonies on the agar surface in the lowest concentration of sample was defined as the minimum bactericidal concentration (MBC). Each assay in this experiment was replicated three times.

### Mice infection model

Mice were fed a basal diet for one week before the study. On treatment Day 0, they were randomly allocated into 14 groups of 10 mice. Herbal extracts and TCM formulas were dissolved in water and given orally at 5 mg/mouse (250 mg/kg) for seven days by a feeding tube. Control and water groups were given a basal diet and fed by water instead. After the last dose, each herbal-treated and watered mouse was challenged orally with 10^10^ CFU of strain ST21. Clinical signs and body weight were monitored, and feces from all mice were collected daily for four days post-challenge. All livers, sera, intestines, and spleens were collected on Day 4 post-challenge, after being humanely euthanized by isofluorane overdose.

### Shedding of challenge bacteria from feces

Fecal samples were collected at 0–4 days after administering strain ST21, and the numbers of *Salmonella* per gram in the feces were determined. Aliquots (100 µl) of fecal suspensions were serially diluted in PBS, then plated on duplicate *Salmonella*-*Shigella* (SS) agar plates (Difco, NJ) and incubated overnight at 37°C. Typical colonies were counted for plates containing 30–300 colonies. *S.* Typhimurium was confirmed by PCR assay, as described in a previous report [21].

### Measurement of bacterial load in blood and organs

After the sacrifice, mouse livers, spleens, intestines were aseptically removed. Heart blood was taken directly from the heart via microsyringe to determine CFU counts, cytokine, and *S.* Typhimurium specific antibody detection. Organs were homogenised in 1.0 ml of sterile saline with aid of a tissue homogeniser maintained at 4°C. Aliquots of the homogenate were then processed for CFU counts and cytokine detection.

### Enzyme-linked immunosorbent assay (ELISA) for evaluation of cytokine levels

Tumor necrosis factor-alpha (TNF-α) and interferon-gamma (IFN-γ) in peripheral blood and intestinal tissue homogenates were detected with an ELISA kit (BioLegend, Inc., San Diego, CA) according to instructions of manuals.

### Detection of *S.* Typhimurium specific IgA and IgM

Anti-ST21-specific antibodies, including immunoglobulin M (IgM) and A (IgA) in mice, were detected by ELISA in sera collected four days post-treatment. The 96-well plates were coated with 100 µl/well of heat-killed ST21 harvested from a bacterial culture (1×10^11^ CFU/ml) at 1∶100 dilution in carbonate/bicarbonate coating buffer (pH 9.6) at 4°C overnight. Coated plates were first blocked with bovine serum albumin (BSA) buffer (1% of BSA in PBS) at room temperature for 2 hr, then washed 5 times in PBS containing 0.1% Tween 20 (PBST). 100 µl/well of mouse serum diluted 1∶10 in BSA buffer was then was added to the coated plates, which were incubated for 2 hr at room temperature. After washing as above, 100 µl/well of peroxidase-conjugated goat anti-mouse IgA or IgM antibody (KPL, London, UK) diluted 1: 5000 in BSA buffer was added to each well after which, the plates were incubated at room temperature for 1 hr. After 5 washings as above, 100 µl/well of tetramethylbenzidine (dissolved 1 mg/ml in DMSO) (KPL, London) was added. Following incubation for 30 min at room temperature, 100 µl/well of stop solution (2 N N_2_SO_4_) was added and the optical density (OD) at 450 nm of each well was measured.

### Preparation of the serum metabolites of CR and SJZT

The serum metabolites of CR or SJZT were prepared from rats, to mimic the molecular forms *in vivo*. CR (2 g/kg) or SJZT (2 g/kg) were orally administered to male Sprague–Dawley rats after overnight fasting. Half an hour later, rats were boosted with another dose of CR or SJZT. Twenty minutes after the second booster, their blood was collected via cardiopuncture. After coagulation, and centrifuging at 10,000 g for 15 min, sera were collected and divided into aliquots and stored at −80°C for later use. In addition, blank serum was processed likewise as a control for comparison to correspondent specimens of CR or SJZT serum metabolites.

### Cell viability assay

Murine monocyte/macrophage cell line RAW 264.7 (ATCC TIB-71) was obtained from ATCC. RAW264.7 cells were seeded onto 24-well plates at 1×10^5^ cells/well for 24 hr in Dulbecco's Modified Eagle Medium (DMEM) supplemented by a 10% fetal bovine serum (FBS). Designated concentrations of berberine, ginsenoside Rb1, or glycyrrhizin were then added to cells, while only adding 0.1% DMSO in the control group, and grown at 37°C for 3.5 hr. The trypan blue exclusion protocol ascertained cell viability. In brief, about 10 µl of cell suspension in PBS (pH 7.4) was mixed with 10 µl of trypan blue, then stained (dead) and unstained (live) cells counted by a hemocytometer. Viability represented the percentage of cell survival after treatment.

### Invasion assay

RAW264.7 cells were treated with serum metabolites, berberine, ginsenoside Rb1, or glycyrrhizin for 30 min before being infected in antibiotic-free DMEM supplemented by 10% FBS, then cocultured in PBS-resuspended ST21 at a multiplicity of infection (MOI) of 10. Designated concentrations of each indicator compound were then added to cells, while only adding 0.1% DMSO to the infection group. Serum was heat-inactivated by incubation at 56°C for 30 min through culture medium as indicated concentration. Cell-associated bacteria were quantified 30 min after infection, then treated with 100 µg/ml of gentamicin for 1.5 hr. Cell culture supernatants were removed gently, then the cells washed with PBS, and osmotically lysed to quantify total bacteria. For this purpose, sterile water was added to infected cells after washing and the cell lysates resuspended with PBS; then bacterial numbers were derived by plating serial dilutions on SS agar plates. The invasion activity was calculated as a triplicate mean, with the results expressed as a percent relative to the invasion of RAW264.7 cells compared to the infection group.

### Detection of TNF-α levels in infected cells

RAW264.7 cells were treated with serum metabolites, berberine, ginsenoside Rb1, or glycyrrhizin for 30 min before being infected in antibiotic-free DMEM supplemented by 10% FBS, then cocultured in PBS-resuspended ST21 at MOI 10. Designated concentrations of each indicator compound were then added to cells, while only adding 0.1% DMSO to the infection group. Serum was heat- inactivated by incubation at 56°C for 30 min and then diluted with culture medium at the indicated concentrations. TNF-α levels were quantified 3 hrs after infection by using ELISA kits as previous described. Results were calculated as triplicate mean and expressed as a fold compared with control group.

### Statistical analysis

Differences between treated and control groups in mean values were rated by Student's t-test, using SPSS software (SPSS, Inc., Chicago, IL).

## Results

### 1. Antibacterial activity of herbal extracts and TCM formulas

In order to identify herbs with antimicrobial properties against *S*. Typhimurium infection, six medicinal herb plants and four TCM formulas were tested. The MBCs of all herbs and formulas against *S*. Typhimurium strains were summarized in Table 3. CR was the only one found under a concentration of 50 mg/ml to have antibacterial activity when tested against the *Salmonella* strains. The MBCs of CR against the reference strain and ST21 were 12.5 mg/ml and 25 mg/ml respectively. Thus, CR showed moderate antibacterial activities against *S*. Typhimurium *in vitro*, and the reference strain was more susceptible to CR than the clinical isolate.

**Table 3:** MBCs of herbal extracts, TCM formulae, and compounds against *S*. Typhimurium strains.

Herbal extract	ATCC 6994	Strain ST21
*Coptidis rhizome*	12.5 mg/ml	25 mg/ml
*Houttuyniae herba*	>50 mg/ml	>50 mg/ml
*Taraxaci herba*	>50 mg/ml	>50 mg/ml
*Glycyrrhizae radix*	>50 mg/ml	>50 mg/ml
*Puerariae radix*	>50 mg/ml	>50 mg/ml
*Rhizoma dioscoreae*	>50 mg/ml	>50 mg/ml
Huoh Shiang Jeng Chih San	>50 mg/ml	>50 mg/ml
Ger Gen Chyn Lian Tang	>50 mg/ml	>50 mg/ml
Shenling Baizhu San	>50 mg/ml	>50 mg/ml
Si Jun Zi Tang	>50 mg/ml	>50 mg/ml
Berberine	5 mM	5 mM
Ginsenoside Rb1	>10 mM	>10 mM
Glycyrrhizin	>10 mM	>10 mM

### 2. Effects of herbal extracts and TCM formulas in preventing *S.* Typhimurium infection in a murine model

**Figure 1:**
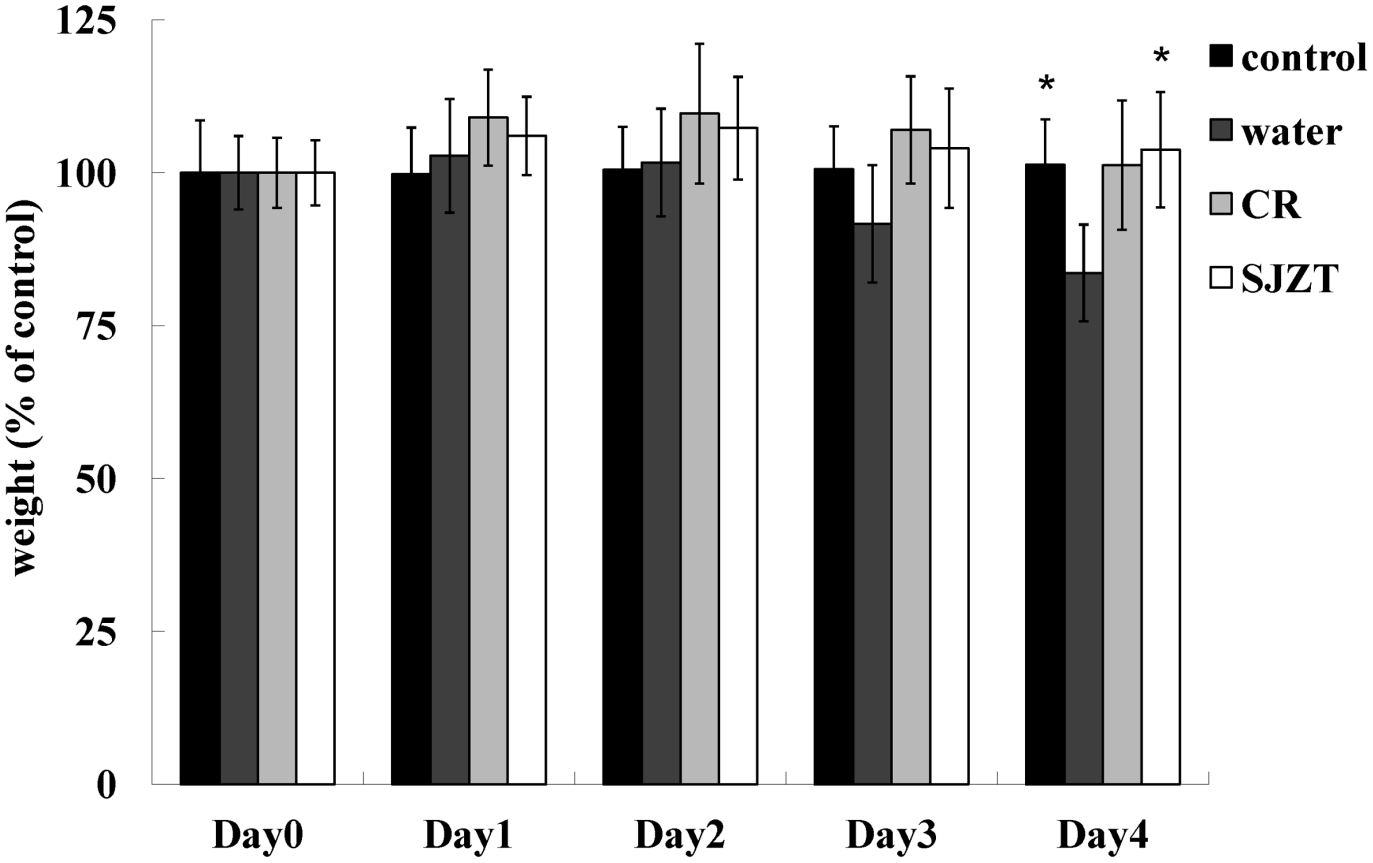
Effects of CR and SJZT supplements on body weight of infected mice. Body weight of the control group was set as 100%, results expressed as mean ± standard deviation (10 mice per group). Asterisks indicate significant difference from the water group collected the same day (*P<0.05).

**Figure 2:**
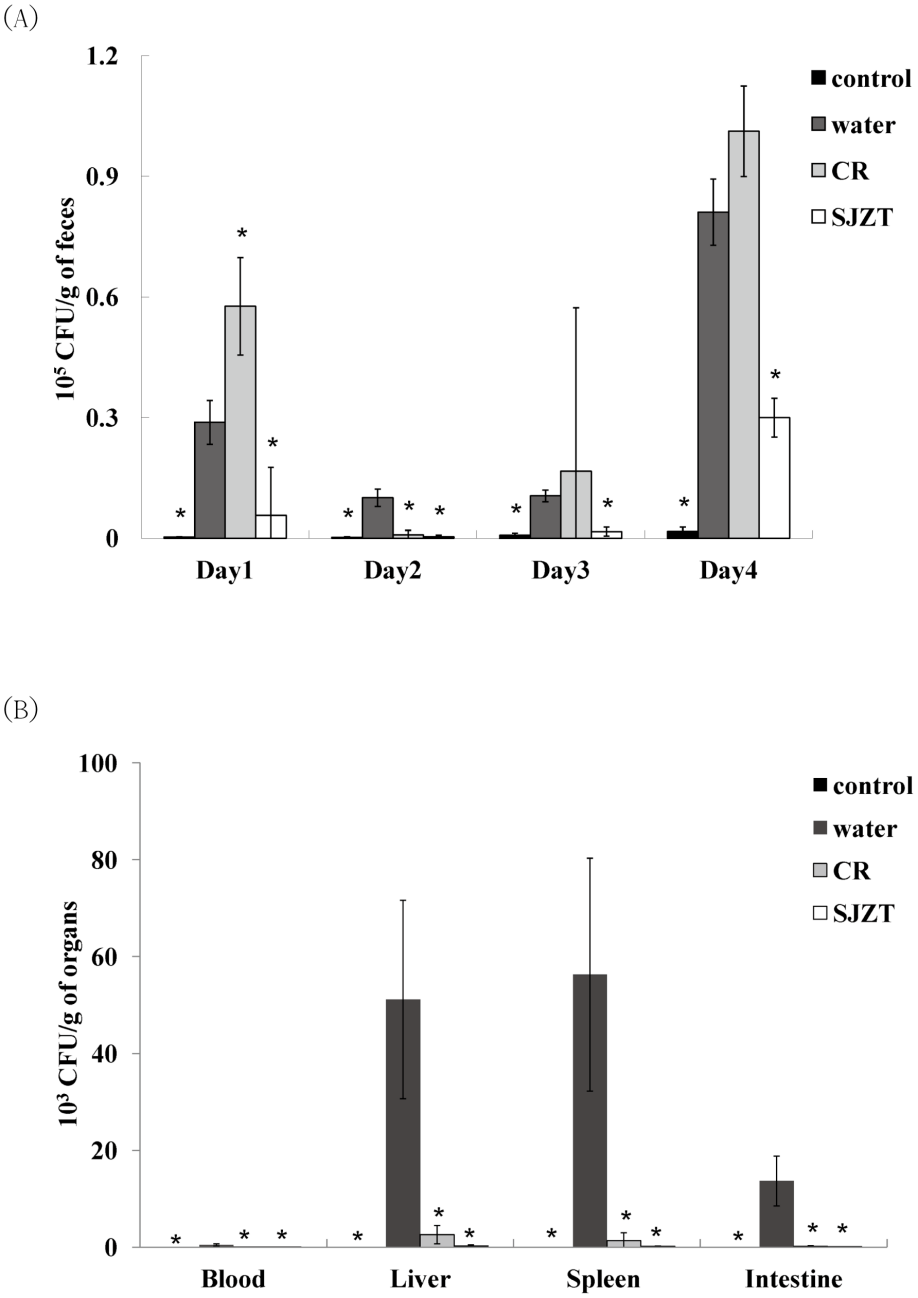
Effects of CR and SJZT supplements on bacteria load in (A) feces and (B) blood, livers, spleens, and intestines on fourth day post-challenge. Results are expressed as mean ± standard deviation (10 mice per group) Asterisks indicate significant difference from the water group (*P<0.05).

**Figure 3:**
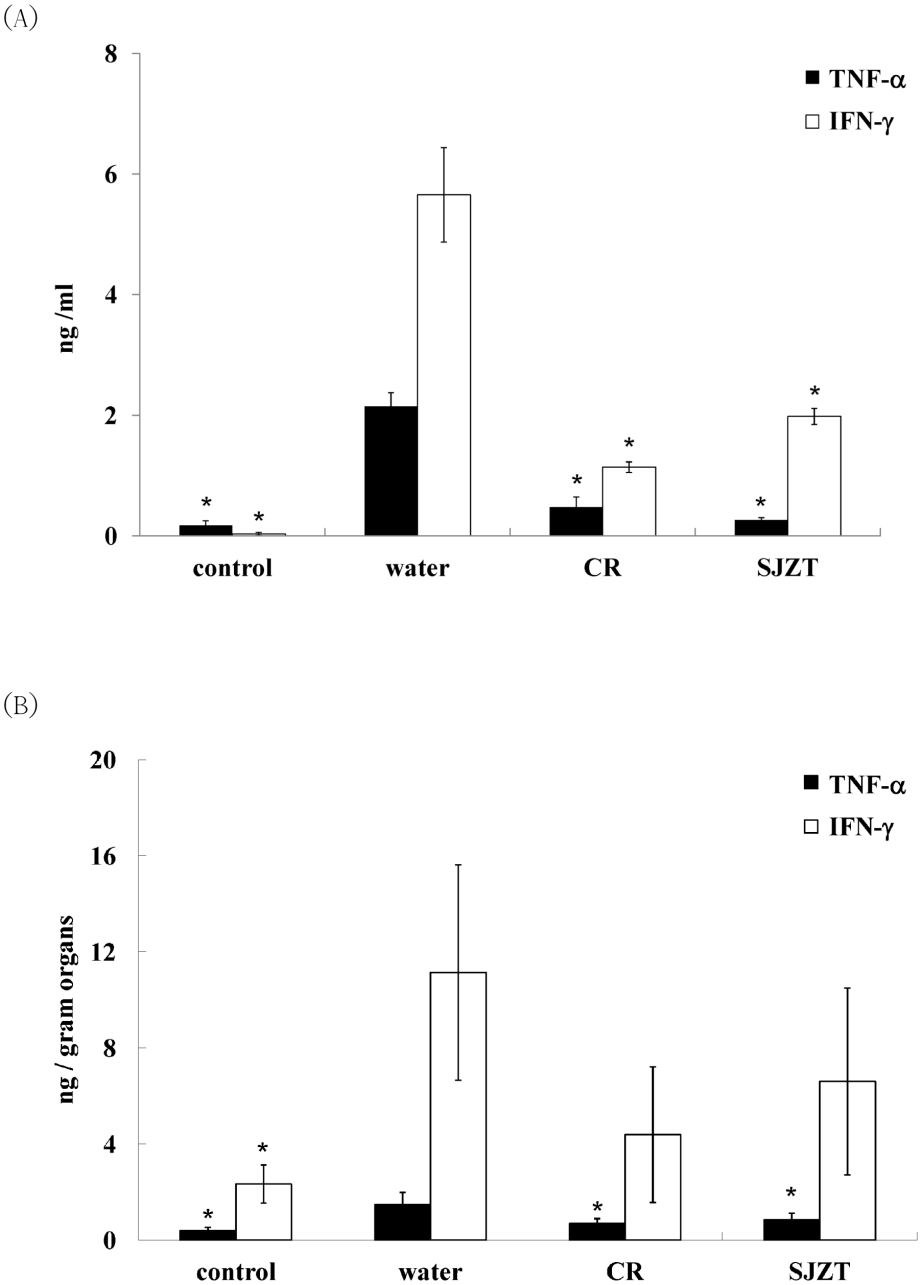
Effects of CR and SJZT supplements on ST21-induced TNF-α and IFN-γ expression in (A) serum and (B) intestines on Day 4 post-challenge. Results are expressed as mean ± standard deviation (10 mice per group). Asterisks indicate significant difference from the water group (*P<0.05).

**Figure 4:**
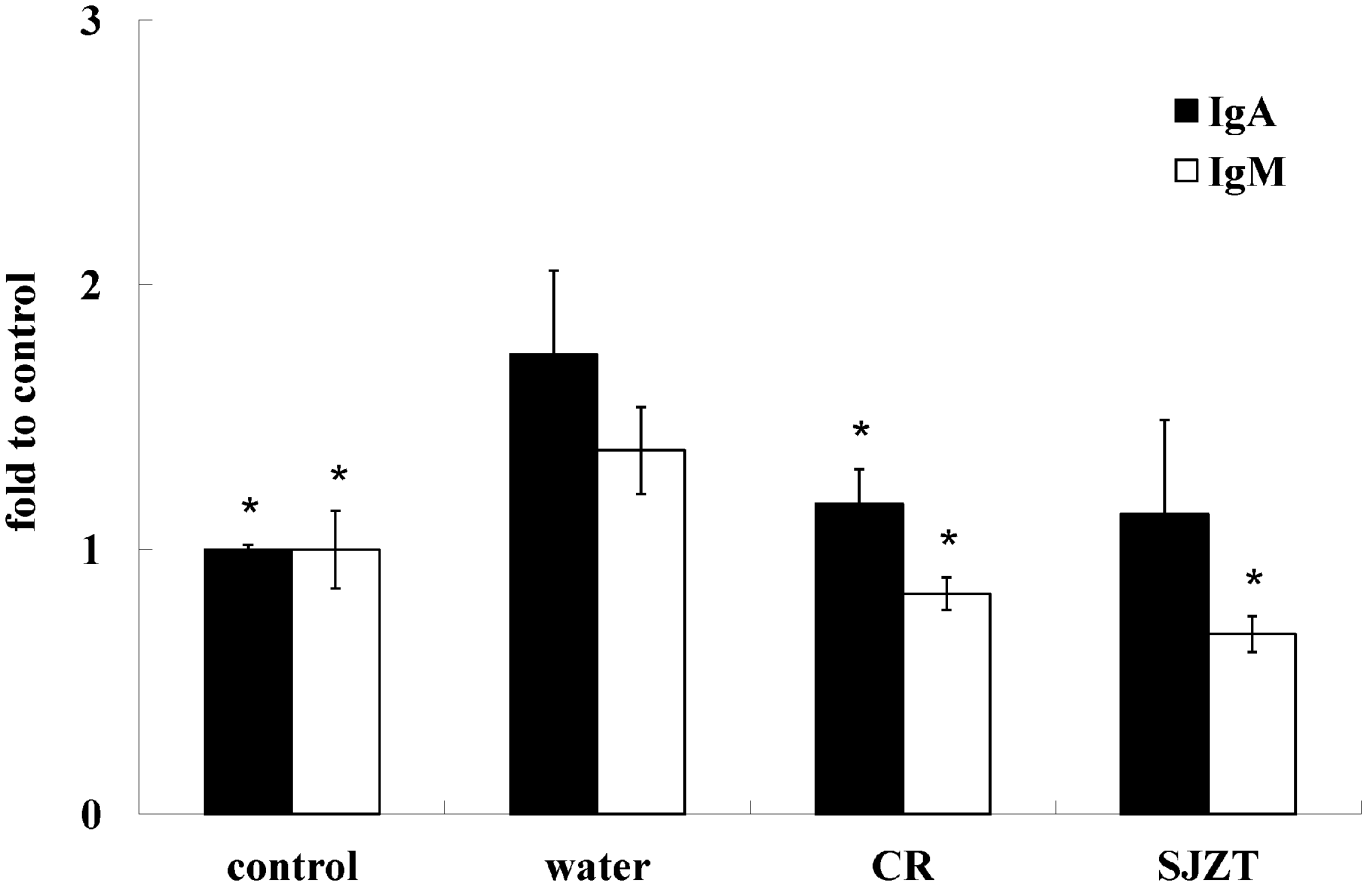
Effects of CR and SJZT supplements on ST21-induced IgM and IgA levels in serum on Day 4 post-challenge. IgM and IgA levels of the control group were set as 100%, results expressed as mean ± standard deviation (10 mice per group). Asterisks indicate significant difference from the water group (*P<0.05).

The supplement dosage of herbal medicine was 5 mg/mouse: i.e., 0.1% of everyday mouse diet [22] was used. After a seven-day treatment, mice were challenged with a single dose of isolate ST21, clinical signs and body weight were monitored. ST21 infection caused mild diarrhea in BALB/c mice. All herbal plants could alleviate diarrhea, but only SJZT could further suppress weight loss resulting from the challenge (Fig. 1) and the bacterial load in feces (Fig. 2A) on Day 4. CR and SJZT reduced bacterial load in blood, livers, spleens, and intestines on Day 4 (Fig. 2B). Other herbal extracts and formulae showed insignificant effects on bacterial shedding or body weight loss (data not shown). Levels of IFN-γ and TNF-α were monitored in infected mice. Only CR and SJZT significantly decreased *S*. Typhimurium-induced IFN-γ levels in serum by 79.84% and 64.95%, respectively. Serum TNF-α also fell 79% and 89% by CR and SJZT, respectively (Fig. 3A), and cytokine levels in the intestinal tissue homogenates were affected by such extracts as well. The CR- and SJZT-treated mice showed an averaged 53.41% and 42.45% decrease of TNF-α (Fig. 3B) respectively, meaning CR and SJZT suppressed *Salmonella*-induced inflammation, unlike the other herbs and formulae which did not. Levels of IgA and IgM in serum were induced by the ST21 infection in our model (Fig. 4). Only CR and SJZT decreased infection-induced IgM level by about 50%; CR also suppressed serum IgA level by 41%. Thus, CR and SJZT could suppress the bacteria load as well as humoral immune response against *Salmonella*.

### 3. Effects of CR and SJZT Metabolites in preventing *S.* Typhimurium invasion and TNF-α expression in infected RAW264.7 cells

In order to further confirm the therapeutic effects of CR and SJZT, the serum metabolites of CR and SJZT were prepared to mimic the *in vivo* conditions. Their effects on the infected RAW 264.7 cells were investigated. Since a larger volume of blood could be collected from rats, rat serum was used in the *in vitro* tests. The dosage used in rats was 16 times higher than in mice. Thus, we were able to investigate the dose effect of metabolites. Three dosages were tested, 4×, 2×, and 1×, which indicated a 4-, 8-, and 16-fold dilution of rat serum. We considered the concentration of metabolites in 1× group to be equal to the concentration in the previous mice serum, the 2× group was equal to twice condensed, and so on.

**Figure 5:**
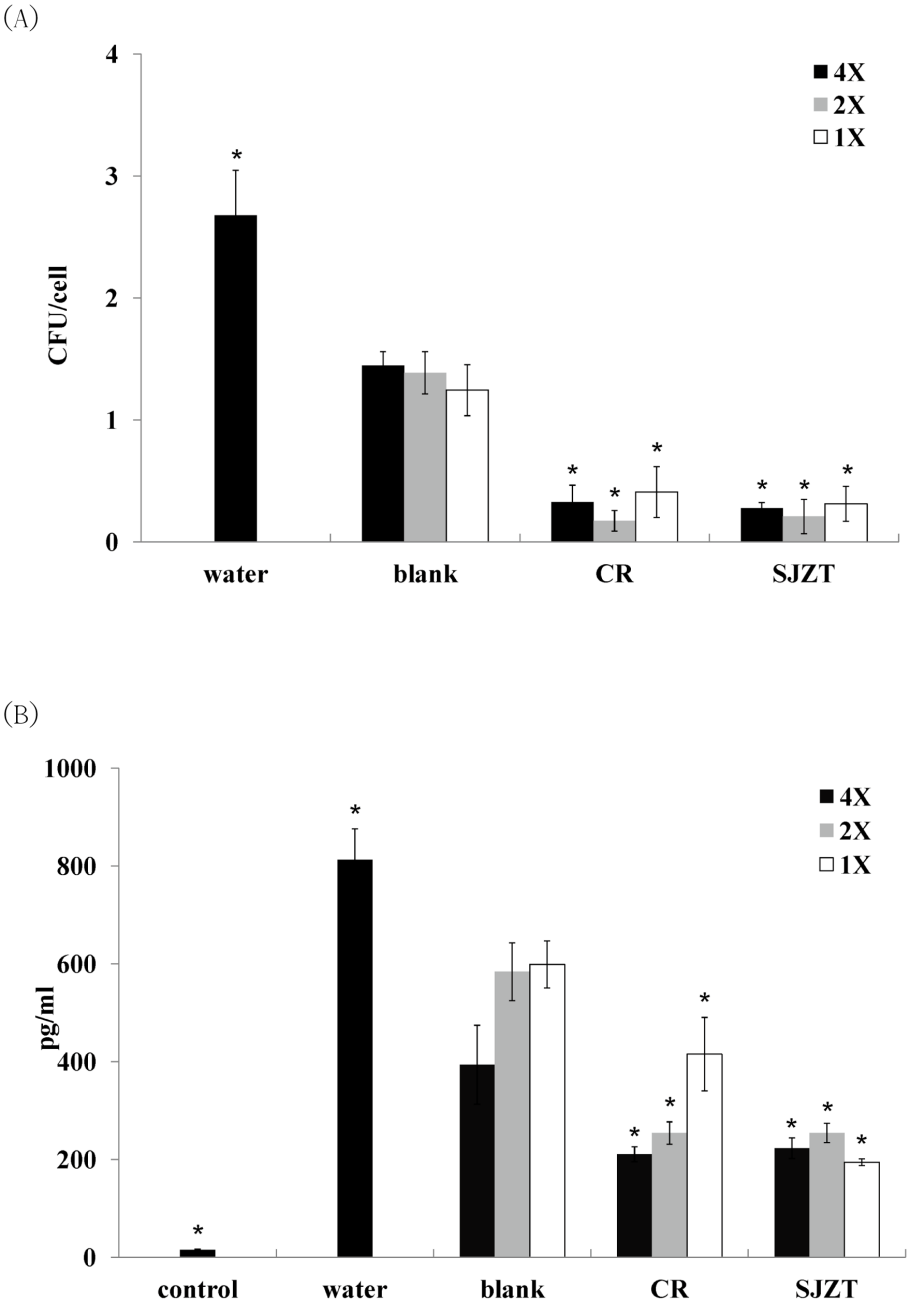
Effects of CR and SJZT Metabolites on (A) ST21 invasion and (B) TNF-α expression in infected RAW264.7 cells. Blank was untreated serum. 4X, 2X and 1X groups were 4-, 8-, and 16-fold dilutions of rat serum. Results are expressed as mean ± standard deviation from three independent experiments. Asterisks indicate significant difference from the blank group (*P<0.05).

Serum metabolites of CR and SJZT significantly reduced the invasion of ST21 in macrophages (Fig. 5A). When compared to the blank group (serum collected from untreated rats), both metabolites could suppress 80% of invasion. This data suggested that the 16-fold diluted serum metabolites were sufficient in affecting the binding efficiency of ST21. Even though we had already heat-inactivated the serum, certain nonspecific antibodies might still interfere with the binding of ST21. Therefore, it still showed a 50% suppression of invasion in the blank group.

TNF-α expression in RAW264.7 cells was also decreased by the serum metabolites of CR and SJZT (Fig. 5B). Compared with blank group, serum metabolites could reduce 30.6% to 67.6% of TNF-αlevels induced by infection. These data also indicated that 16-fold diluted SJZT serum metabolite was sufficient to reduce ST21-induced TNF-α expression.

### 4. Effects of berberine, ginsenoside Rb1, and glycyrrhizin in preventing *S.* Typhimurium invasion and TNF-α expression in infected RAW264.7 cells

It was revealed that berberine and glycyrrhizin—two of the indicator compounds—were present in the serum metabolites of CR and SJZT, respectively [23], [24] along with the third indicator compound ginsenoside Rb1 (Table 2). Therefore, the bioactivities of these three were further assessed *in vitro*.

**Figure 6:**
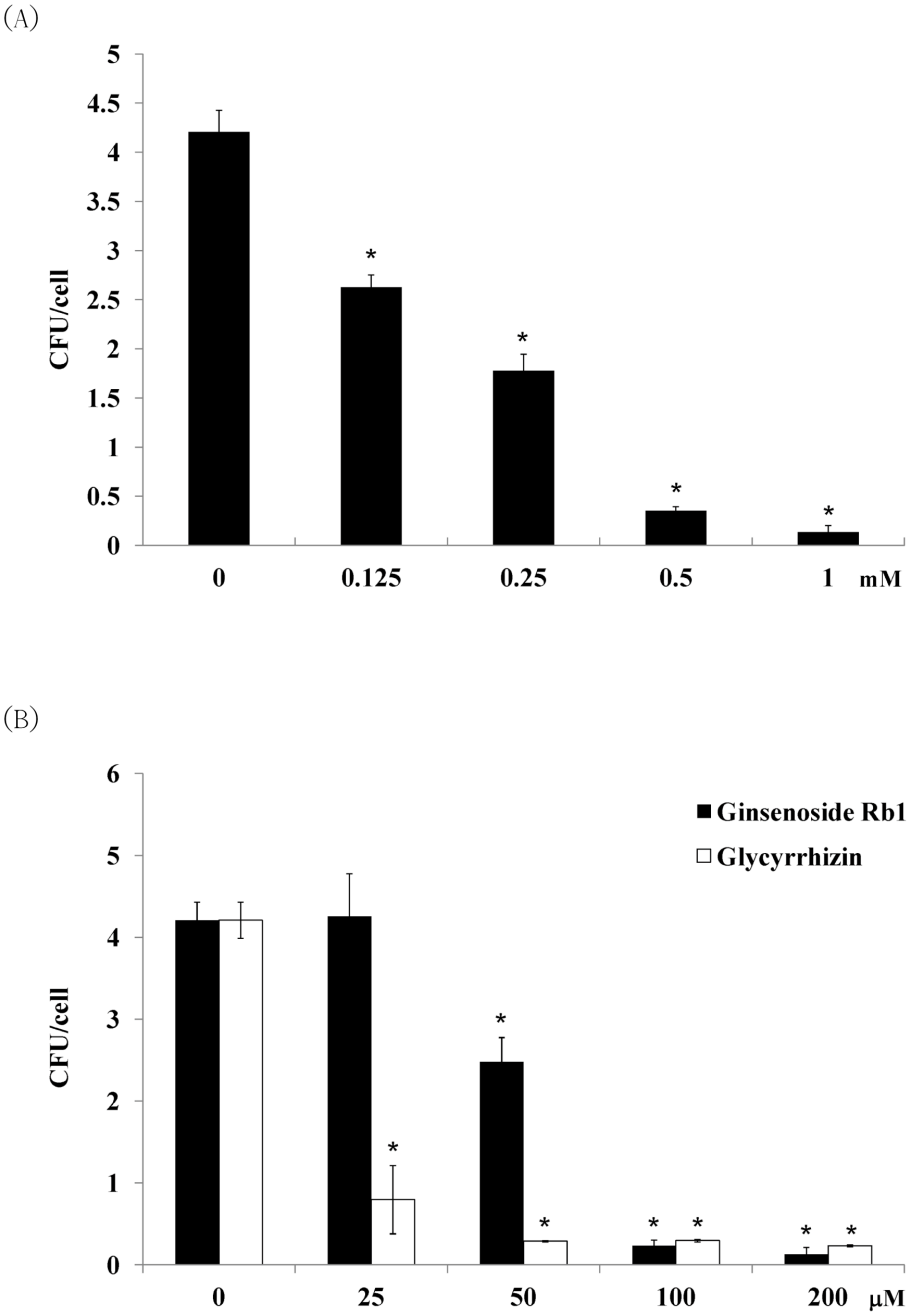
Effects of (A) berberine, (B) ginsenoside Rb1, and glycyrrhizin on ST21 invasion in infected RAW264.7 cells. Results are expressed as mean ± standard deviation from three independent experiments. Asterisks indicate significant difference from the water group (*P<0.05).

**Figure 7:**
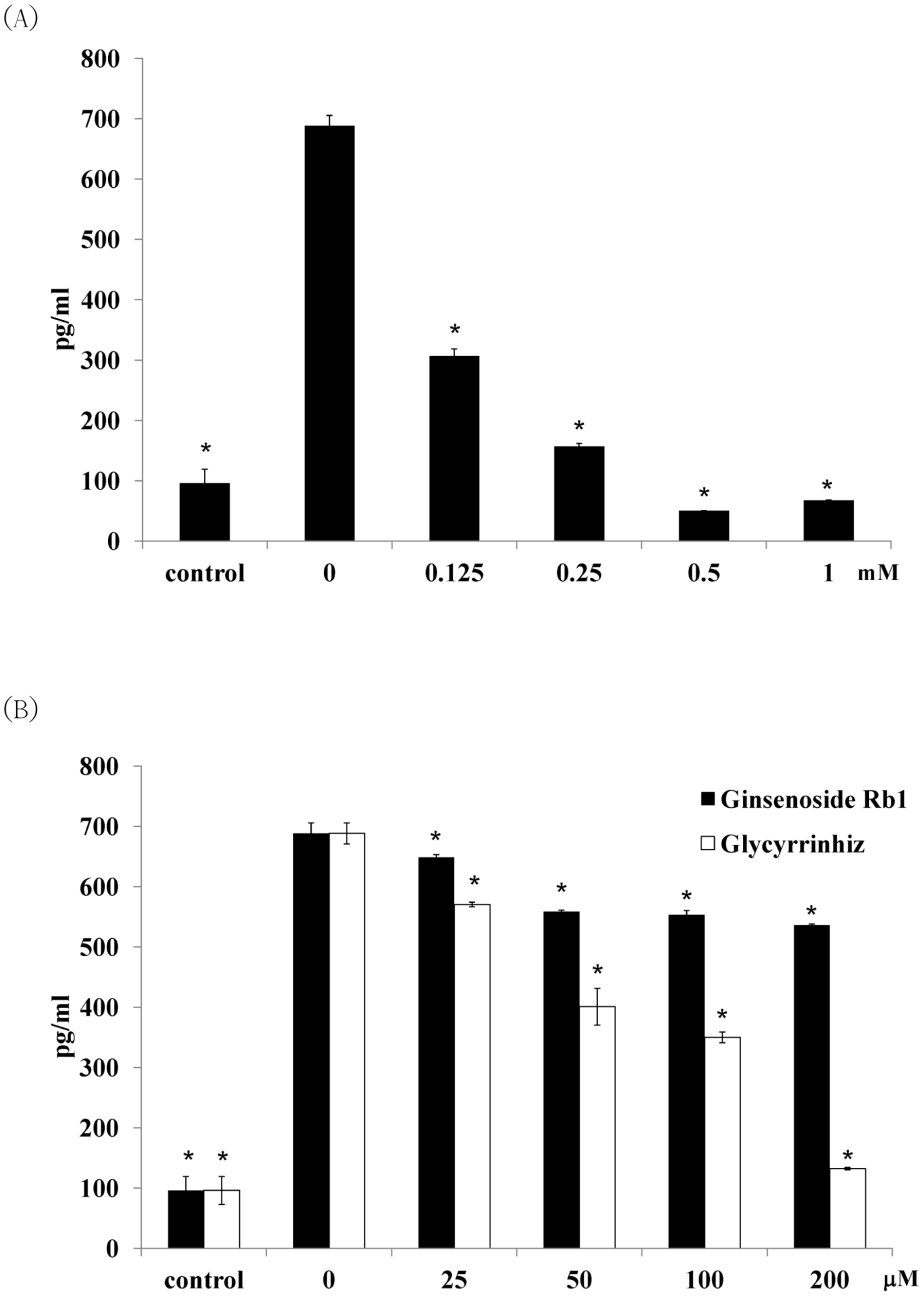
Effects of (A) berberine, (B) ginsenoside Rb1, and glycyrrhizin on ST21 TNF-α expression in infected RAW264.7 cells. Results are expressed as mean ± standard deviation from three independent experiments. Asterisks indicate significant difference from the water group (*P<0.05).

MBCs of berberine to both *Salmonella* strains were 5 mM (Table 3). However, bactericidal activities of ginsenoside Rb1 and glycyrrhizin rose higher than 10 mM. IC_50_ of berberine for RAW264.7 cells were 1.3 mM at 3.5 hr. However, the cell viability was not affected by less than 3 mM of ginsenoside Rb1 and glycyrrhizin. The abilities of these compounds to prevent a ST21 infection of macrophages were studied by an invasion assay (Fig. 6A). By pretreating macrophages with indicator compounds for 30 min, 1 mM of berberine prevented 97% of bacteria from invading cells (Fig. 6A); 100 µM of ginsenoside Rb1 suppressed 94%. Still, glycyrrhizin suppressed 81% of bacterial invasion at a concentration of 25 µM (Fig. 6B). The three indicator compounds thus showed capacity in preventing *Salmonella* from invading macrophages in a dose-dependent manner. Glycyrrhizin showed the best ability in blocking bacteria invasion, followed by ginsenoside Rb1. Along with the invasion ability, the expression of TNF-α induced by infection was suppressed simultaneously by the treatment—also in a dose-dependent manner (Fig. 7).

## Discussion

Our mouse model gauged the efficacy of six herbal plants and four TCM formulas in preventing *S.* Typhimurium infection, with only CR showing moderate bactericidal activities *in vitro*. In the mouse model, CR and SJZT has efficiently decreased the bacterial shedding in feces and the bacterial load in the organs and blood, hinting at the bioactivity of herbal materials being converted in organisms, which is difficult to evaluate *in vitro*. We indicated that, while bactericidal activities of the indicator compounds of CR and SJZT (berberine, ginsenoside Rb1, and glycyrrhizin) were ineffective, however, they still prevented *Salmonella* from invading macrophages and hence might play a role in suppressing bacteria spreading systemically via circulation *in vivo*.

Interaction between compounds during preparation *in vitro* or metabolism *in vivo* might complicate the efficiency of their bioactivities. SJZT is a well-known formula that can modify the immunity in humans [25]. In this mouse model, only the TCM formula SJZT could suppress body weight loss caused by infection. The ingredients of SJZT might interact with each other *in vivo*, causing this adjustment in immunity and eliminating the effects of infection.

In this study, we investigated the effects of the serum metabolites of CR and SJZT on preventing *Salmonella* invasion. Both of them significantly decreased the invasion and TNF-α expression induced by infection. These data indicated that the active ingredients of CR and SJZT that prevents an infection were metabolized and present in the serum. Based on our *in vitro* results, berberine, ginsenoside Rb1, and glycyrrhizin might be the candidates.

A *Salmonella* infection could trigger pro-inflammatory response in both infected and neighboring cells [26]. In our study, the bacterial load in the organs was decreased by CR and SJZT treatment; thus ST21-induced TNF-α expression was decreased in the intestinal tissues. A *S*. Typhimurium infection could also activate macrophages through IFN-γ in order to trigger production of nitric oxide as a host defense [27]. Since berberine, ginsenoside Rb1, and glycyrrhizin prevent bacteria from invading macrophages, CR and SJZT can lower IFN-γ level in serum of infected mice. In summary, if CR and SJZT are in the diet, there will be a significantly decreased *Salmonella* load in the organs and blood while also decreasing both TNF-α and IFN-γ levels in serum, as well as the TNF-α levels in the intestinal tissue caused by infection.

Since *Salmonella* can penetrate the epithelial lining of the intestines, an IgA response would then develop [28]. IgM appears early in the process of infection. CR could suppress both serum IgA and IgM levels in infected mice, but SJZT could only decrease IgM levels—a detailed mechanism that needs further investigation.

Both CR and SJZT did not show strong bactericidal activity, nor did berberine, ginsenoside Rb1, and glycyrrhizin. The mechanism of preventation might involve modulating the immunity against the infection, which is in a more systemic aspect. Herbal extracts consist of a variety of compounds with diverse characteristics and hence offer an alternative way to prevent infection. It has been shown that berberine, ginsenoside Rb1, and glycyrrhizin have immunomodulating effects. Berberine could reduce the inflammatory responses caused by inflammatory bowel disease (IBD) [29]; ginsenoside has been shown to inhibit viral influenza induced inflammation [30]; glycyrrhizin has also revealed its way of modulating the innate immune responses mediated by Toll-like receptors (TLRs) [31]. Therefore, CR and SJZT has shown their potential as preventive agents.
